# Supplementary material for: Sex-specific expression profiles of ecdysteroid biosynthesis and ecdysone response genes in extreme sexual dimorphism of the mealybug Planococcus kraunhiae (Kuwana)
Source: PLoS One. 2020 Apr 13;15(4):e0231451. doi: 10.1371/journal.pone.0231451 (PMC7153872; doi:10.1371/journal.pone.0231451)
Supplement: S1 Fig — Protein sequences of Spook (A), Disembodied (B), Shadow (C) and Shade (D) were aligned among Planococcus kraunhiae (Pk), Bombyx mori (Bm), Drosophila melanogaster (Dm) and Tribolium castaneum (Tc). Putative “PERF” domains as well as heme-binding domains were indicated with lines. Accession numbers follow gene names. (PDF) [file pone.0231451.s001.pdf]

S1 Fig

A

```
PkSpo                      ------------------------------------------------------------
BmSpo_NP_001104833.1       ------------------------------------------------------------
DmSpo_NP_647975.2          ------------------------------------------------------------
TcSpo_EFA11558.2           MGRWVWSRAECGSSGDCLEEVSKSAVCARGPITDDLMCLKMMTFGLAKCTITGVDGHGPR 60


PkSpo                      ------------------------------------------------------------
BmSpo_NP_001104833.1       -MSSLIIVFFVFALAVYKL--------LRRKTERWVKTNKYGGVETAILR---------- 41
DmSpo_NP_647975.2          MLAALIYTILAILLSVLATSYICIIYGVKRRVLQPVKTKNSTEINHNAYQK--------- 51
TcSpo_EFA11558.2           DGGTPILLVAGAATGHLQANLQIAAAPPGGAREGSCHWSRTNSVNLSVLGADMLALLCLC 120


PkSpo                      ------------------------------------------------------------
BmSpo_NP_001104833.1       ----------------TAPGPVCWPIIGSLHLLGGH-ESPFQAFTELSKKYGDIFSVKLG 84
DmSpo_NP_647975.2          --------------YTQAPGPRPWPIIGNLHLLDRYRDSPFAGFTALAQQYGDIYSLTFG 97
TcSpo_EFA11558.2           VVLLVWWFSRPKKSPSTIPGPRPWPLIGSMHLLAGH-ETPFQAFTALSRVYGDIFSIHLG 179


PkSpo                      ------------------------------------------------------------
BmSpo_NP_001104833.1       SADCVVVNNLSLIREVLNQNGNVVAGRPDFLRFHKLFAGDRNNSLALCDWSNLQLRRRNL 144
DmSpo_NP_647975.2          HTRCLVVNNLELIREVLNQNGKVMSGRPDFIRYHKLFGGERSNSLALCDWSQLQQKRRNL 157
TcSpo_EFA11558.2           SASCVVVNNFKLIKEVLIAKGGDFGGRPDFARFHKLFGGDRNNSLALCDWSSLQKTRRSI 239


PkSpo                      -------------------------------------------------------MFTQY 5
BmSpo_NP_001104833.1       ARRHCGPKQHTDSYARIGTVGTFESVELIQTLKGLTSRSDASIDLKPILMKSAMNMFSNY 204
DmSpo_NP_647975.2          ARRHCSPREFSCFYMKMSQIG-CEEMEHWNRELGNQLVPGEPINIKPLILKACANMFSQY 216
TcSpo_EFA11558.2           ARTYCSPRFTSLQYDRVNNVGEEELKSFLHQLD--QLPHGQPCNVKPAVLMVCANMFTQY 297
                                                                                  **::*

PkSpo                      MCTVRFPYDLKSFQNIVRYYDEVFWDINQGYAVDFMPWLSPLYYRHMNKLSWWAGEVRKF 65
BmSpo_NP_001104833.1       MCSVRFDDEDLEFQKIVDHFDEIFWEINQGYAVDFLPWLAPFYKKHMEKLSNWSQDIRSF 264
DmSpo_NP_647975.2          MCSLRFDYDDVDFQQIVQYFDEIFWEINQGHPLDFLPWLYPFYQRHLNKIINWSSTIRGF 276
TcSpo_EFA11558.2           MCSTSFAYEDKGFQKIVRYFDEIFWEINQGYAVDFLPWLLPVYTGHMKKISNWATEIRQF 357
                           **:  *  :   **:** ::**:**:****:.:**:*** *.*  *::*:  *:  :* *

PkSpo                      IMQHIVNEHLSTIDYEEPPRDFTDALLQNLKTDSQLTWQHIMFELEDFLGGHSAVGNLVM 125
BmSpo_NP_001104833.1       ILSRIVEQREISLDTEAPEKDFLDGLLRVLHEDPTMDRNTIIFMLEDFLGGHSSVGNLVM 324
DmSpo_NP_647975.2          IMERIIRHRELSVDLDEPDRDFTDALLKSLLEDKDVSRNTIIFMLEDFIGGHSAVGNLVM 336
TcSpo_EFA11558.2           ILSRIIDKHRATLDTNSPPRDFTDALLMHLEEDPNMNWQHIIFELEDFLGGHSAIGNLVM 417
                           *:.:*: .:  ::* : * :** *.**  *  *  :  : *:* ****:****::*****

PkSpo                      LTLVSLVKYPYVKKRIEEEIQTVTGGS-RNVGMLDKTEMVYTEATVFETLRVTSSPIVPH 184
BmSpo_NP_001104833.1       LCLTAVARDPEVGRKIRQEIDAVTRGK-RPVGLTDRSHLPYTEATILECLRYASSPIVPH 383
DmSpo_NP_647975.2          LVLAYIAKNVDIGRRIQEEIDAIIEEENRSINLLDMNAMPYTMATIFEVLRYSSSPIVPH 396
TcSpo_EFA11558.2           VTLAAVVDHPEVAKRIQEEVDQVTGGT-RCPNLFDKAAMPYTEATILETLRTASSPIVPH 476
                           : *. :.    : ::*.:*:: :     *  .: *   : ** **::* ** :*******
                                                                      PERF motif
PkSpo                      VATQDTELGGHFIEKGTCVILNNFELNKSCEYWEKPTEFLPERFIK-------------- 230
BmSpo_NP_001104833.1       VATENANISGYGIEKGTVVFINNYVLNNSEQYGSEPEKFDPSRFLEKTRVRTRRNSQ-CD 442
DmSpo_NP_647975.2          VATEDTVISGYGVTKGTIVFINNYVLNTSEKFWVNPKEFNPLRFLEPSKEQSPKNSKGSD 456
TcSpo_EFA11558.2           VASKDTEIDGHEVSKGTIVFINNYELNQGDAYWDEPGLFKPERFLSS------------- 523
                           **:::: :.*: : *** *::**: **  .  :  :*  * * **:.
                                                                   Heme-binding
PkSpo                      -------------------DGKVVKP--AHFIPFGTGKRTCIGQQLVSGFSFVLVAGIVQ 269
BmSpo_NP_001104833.1       SGLESDSERAPVGKPDVEREMLSVKKNIPHFIPFSIGKRTCIGQTMVTSMSFTMFANIMQ 502
DmSpo_NP_647975.2          SGIESDNE------------KLQLKRNIPHFLPFSIGKRTCIGQNLVRGFGFLVVVNVMQ 504
TcSpo_EFA11558.2           -------------------TGNIVKP--AHFIPFSTGKRTCIGQRLVQCFSFVVLATLLQ 562
                                                  :*   .**:**. ******** :*  :.* :.. ::*

PkSpo                      HFDIELTTTENLCAKAACVALPPDTFPIVLKTKRKIS-- 306
BmSpo_NP_001104833.1       SFEVGVENINDLRQKPACVALPKNTYKMHLIPRK----- 536
DmSpo_NP_647975.2          RYNISSHNPSTIKISPESLALPADCFPLVLTPREKIGPL 543
TcSpo_EFA11558.2           YYDVS--TKESVKVQPGCVAVPPDCFKLVLTPRK----- 594
                            :::   . . :  .. .:*:* : : : * .:.
```

S1 Fig (continued)

B

```
PkDib                     ------------------------------------------------------------
BmDib_NP_001036953.1      --MFVRLTVKNNIPYRARKCVYRRASENFVGSEHASKVNEQGDNLMNFEDIPGPRSYPII 58
DmDib_AAF60174.1          --MLTK-----LLKISCT--------------------SRQCTFAKPYQAIPGPRGPFGM 33
TcDib_XP_974252.1         MCLFSKKSVHYLLTIRTRNYYYR-----------GRFLTTAVDPPKSFKEIPGPLSLPLV 49


PkDib                     ------------------------------------MIPGLPVLWVFKPEDIEMVYRSEG 24
BmDib_NP_001036953.1      GTLHKYLPLIGDYDAEALDKNAILNWRRYGSLVRE--KPIVNLVHVYDPDDIEAVFRQDH 116
DmDib_AAF60174.1          GNLYNYLPGIGSYSWLRLHQAGQDKYEKYGAIVRETIVPGQDIVWLYDPKDIALLLN-ER 92
TcDib_XP_974252.1         GTLYLYFPFIGRYQFDRLHKNALKNFQLYGPIIREEIVPGEHIVWLGDPDDIAKMFHTEG 109
                                                          *   :: : .*.**  : . :

PkDib                     RYPERRSHLALEKYRLDRPEVYNSGGLLPTNGPEWWRLRKIFQKDLNKIQNVRSYLPKSD 84
BmDib_NP_001036953.1      RYPARRSHTAMNYYRTNKPNVYNTGGLLATNGPDWWRLRSIFQKNFTSPQSVKTHVSDTD 176
DmDib_AAF60174.1          DCPQRRSHLALAQYRKSRPDVYKTTGLLPTNGPEWWRIRAQVQKELSAPKSVRNFVRQVD 152
TcDib_XP_974252.1         TYPYRKSHLTLEKYRLDRPHIYNSGGLLPTNGPEWSRIRKVFQKGLSGPTEALSFIKGSD 169
                            * *:** ::  ** .:*.:*:: ***.****:* *:*  .** :.   .. ..:   *

PkDib                     NVIKDFLRSRISKYKG-----DFLPELDRLYLELTGLVAFDELLGCFSDKEIESGSKMSE 139
BmDib_NP_001036953.1      NIAKEFV--EWIKRDKVSSKNDFLTFLNRLNLEIIGVVAFNERFNSFALSEQDPESRSSK 234
DmDib_AAF60174.1          GVTKEFI--RFLQESRNGGAIDMLPKLTRLNLELTSLLTFGARLQSFTAQEQDPSSRSTR 210
TcDib_XP_974252.1         DVISEWLDTRFKKIHKETSNMDFLQELSRLFLELIGVAAFDIRFQSFHDDELDPCSKSTK 229
                          .: .:::  .  :        *:*  * ** **: .: :*.  : .*  .* :. *: :.

PkDib                     LIDAVAVINGSLLKTDNGPQLWRKFDTPLYKKFCKAHLYLEAIAKDFVRKKMLQLEENPS 199
BmDib_NP_001036953.1      TIAAAFGSNSGVMKLDKG-FLWKMFSTPLYKKLVNSQIYLEKISTDILIRKINLFESDDS 293
DmDib_AAF60174.1          LMDAAETTNSCILPTDQGLQLWRFLETPSFRKLSQAQSYMEGVAMELVEENVR----NGS 266
TcDib_XP_974252.1         LLESAFVTNSTILKTDNGPQLWRKFETPAYRRLRKAQELMESVAIDLVALKLSTFKEKTS 289
                           : :.   *. ::  *:*  **: :.** :::: :::  :* :: :::  ::     . *

PkDib                     AAQLSLLKQYLLNEELDIKDVIGMAADLLLGGIHTMSYTSAFGLYHLSRNMLEQEIMFEE 259
BmDib_NP_001036953.1      KNDKSLLKTFLQQPQLDHKDIMGMMVDILMAAIDTTAYTTSFVLYHIARNKRCQDEMFEE 353
DmDib_AAF60174.1          VGS-SLISAYVKNPELDRSDVVGTAADLLLAGIDTTSYASAFLLYHIARNPEVQQKLHEE 325
TcDib_XP_974252.1         NPP-TLLERYLASASLDFKDIIGVVCDFLLAGMDTTTYSSSFLLYHLATNPSTQDALYEE 348
                              :*:. :: . .** .*::*   *:*:..:.* :*:::* ***:: *   *: :.**

PkDib                     LNSLMT-TNDTITETMLN-QAKYTRAVIKEIFRLSPISVGVGRILAKDAVLSGYFVPSGT 317
BmDib_NP_001036953.1      LHTLLPKKDDEITADVLS-KASYVRSSIKESLRLNPVSIGIGRWLQKDIVLKGYSIPKGT 412
DmDib_AAF60174.1          AKRVLPSAKDELSMDALRTDITYTRAVLKESLRLNPIAVGVGRILNQDAIFSGYFVPKGT 385
TcDib_XP_974252.1         ACRLLPNPAAPLTTEKYK-QAEYAKCAVKESLRLRPISIGVGRQLTTDVVFSGYKVPSGT 407
                             ::.     ::      .  *.:. :** :** *:::*:** *  * ::.** :*.**
                                            PERF motif             Heme-binding
PkDib                     IVVTQNQITCRLPEYFQNPDEFKPERWIRGNPLYKNIHPYLVLPFGHGPRTCIARRLAEQ 377
BmDib_NP_001036953.1      VIVTQNMTSSRLPQFIRDPLTFKPERWMRGSPQYETIHPFLSLPFGHGPRSCIARRLAEQ 472
DmDib_AAF60174.1          TVVTQNMVRCRLEQHFQDPLRFQPDRWLQHRS---ALNPYLVLPFGHGMRACIARRLAEQ 442
TcDib_XP_974252.1         VVVTLNQVLSRMEKYFPEPDSFKPERWMKNDPSYVQTHPYLVIPFGHGQRSCIARRFAEQ 467
                           :** *   .*: :.: :*  *:*:**::  .     :*:* :***** *:*****:***

PkDib                     NFQLFLMNIIRRYKLKWKGD--ELDSISIQINKPDKIVQIEFIER-- 420
BmDib_NP_001036953.1      NICIILMRLIREFEIQWAGE--ELGVKTLLINKPNKPVSLNFIPRSS 517
DmDib_AAF60174.1          NMHILLLRLLREYELIWSGSDDEMGVKTLLINKPDAPVLIDLRLRRE 489
TcDib_XP_974252.1         NMVILILKLARKYKLRWNGS--EIDSKSLLINKPDGPILLSFEPR-- 510
                          *: ::::.: *.::: * *.  *:.  :: ****:  : :.:  *
```

S1 Fig (continued)

C

```
PkSad_variant_B           ------------------------------------------------------------
BmSad_NP_001106224.1      ----------------------------------------------------------MH 2
DmSad_AAL86019.1          -------------------------------------MTEKRERPGPLRWLRHLLDQLLV 23
TcSad_XP_970122.2         MRDGQSSGAINIMPSRISLTASSLNHSVRPDVRLQGETAVEDALGRHPAHGQEAAAADLK 60

PkSad_variant_B           ----MTWKYR--------LPKYSRSYSIDVKSFQEIPSPKGLPYVGTLFSLIISGGANNL 48
BmSad_NP_001106224.1      RFPSMSSIRSAVRSRNSNRCSMSTKPHKSLRTIDEMPHKKSLPIIGTKFDLFSAGGGKNL 62
DmSad_AAL86019.1          RILSLSLFRSRCDPPPLQRFPATELPPAVAAKYVPIPRVKGLPVVGTLVDLIAAGGATHL 83
TcSad_XP_970122.2         FPQAGTMLFRRVLAQLSTRPKSSLVSANTVLHFDEIPSPKGLPLVGTTLALIAHGSTPKL 120
                               :                :            :*   *.** :** . *:  *.  :*

PkSad_variant_B           HRYIDKRHQEFGPIFLEKLG-PITSLFLNDSDEIRKVFM-HEGRYPKHVLPECWLLYNKM 106
BmSad_NP_001106224.1      HKYIDMRHKQLGPIFYERLTGKTKLVFISDPTHMKSLFLNLEGKYPAHILPEPWVLYEKL 122
DmSad_AAL86019.1          HKYIDARHKQYGPIFRERLGGTQDAVFVSSANLMRGVFQ-HEGQYPQHPLPDAWTLYNQQ 142
TcSad_XP_970122.2         HDYVDKRHKQLGPIFKEKLG-PVSAVFVADPDEIRAVFD-HEGKHPLHVLPDAWVAYNQM 178
                          * *:* **:: **** *:*      :*: ..  :: :*   **::* * **: *  *::

PkSad_variant_B           KKYERGLYFMDGEEWFTHRKLLNDMLLKRDYY-SEAEKNEVFVEDMLRRWLSCSDDG--- 162
BmSad_NP_001106224.1      YGSKRGLFFMDGEDWLINRRIMNKHLLREDSDVWLRAPIRTAVFHFICNWKLR------- 175
DmSad_AAL86019.1          HACQRGLFFMEGAEWLHNRRILNRLLLNGNLN-WMDVHIESCTRRMVDQWKRRTAEAAAI 201
TcSad_XP_970122.2         YGCPRGLFFMDGANWWYHRRIMNRLLLKGDFR-WIEGACECVSDKLIDSLMGESD----- 232
                              ***:**:* :*  :*:::*  **. :         .     ::

PkSad_variant_B           -----------KLINLEDELYNLSLIFMMSFVFGN-RFHENTNIFLARIHQLSDIVKDIF 210
BmSad_NP_001106224.1      --AQSGNFS----PNLESEFYRFSTDVILAVLQGNSALLKPTPEYEMLLLLFSEAVKKIF 229
DmSad_AAL86019.1          PLAESGEIRSYELPLLEQQLYRWSIEVLCCIMFGTSVLT--CPKIQSSLDYFTQIVHKVF 259
TcSad_XP_970122.2         -----------YCGNLEATLYKWSLDVIVSILLGSGSYSQLCGELEPKVQKLAQVTHLVF 281
                                         **  :*. *  .: ..: *.             :  ::: .: :*

PkSad_variant_B           EYSVKLSILPATLAMRLNLKIWNSFVNAVDQSVQITRELLHDAIRLYKEN--IDDESILQ 268
BmSad_NP_001106224.1      STTTKLYALPVEFCQRWNLKVWRNFKQSVDDSISIAQKIVYEMLHTKDAG-----DGLVK 284
DmSad_AAL86019.1          EHSSRLMTFPPRLAQILRLPIWRDFEANVDEVLREGAAIIDHCIRVQEDQRRPHDEALYH 319
TcSad_XP_970122.2         QTSAKLALIPASFASKFKIPQWRRFVDSVDNALAQANALVD---TLIEKK--PHSDGLLP 336
                          . : :*  :*  :.   .:  *. *   **: :     ::       .       :.:

PkSad_variant_B           SLLKRKLSTDLIERILVDFILAAGDTTAYTMLWTFYLLGRHKDVQDKLFVDIEQSKRKNE 328
BmSad_NP_001106224.1      RLKDENMSDELITRIVADFVIAAGDTTAYTSLWILFLLSN----NTEILTEMNDND---- 336
DmSad_AAL86019.1          RLQAADVPGDMIKRIFVDLVIAAGDTTAFSSQWALFALSKEPRLQQRLAKERATNDS--- 376
TcSad_XP_970122.2         KLLAEQITLEDIKRIIVDLVLAAGDTTAVAMEWMLYLVAKSPQIQEKLRRNPD------- 389
                           *   .:. : * **..*:::******* :  * :: :..    : .:  :

PkSad_variant_B           QTPAIRNIIKESMRLYPIAPFIARYLPEETFICGYRIPANQLISLSMYNSSRNEKYFPNA 388
BmSad_NP_001106224.1      --QYVKNVVKEAMRLYPVAPFLTRILPKQCVLGPYLLEEGTPVIASIYTSGRDEQNFSKA 394
DmSad_AAL86019.1          --RLMHGLIKESLRLYPVAPFIGRYLPQDAQLGGHFIEKDTMVLLSLYTAGRDPSHFEQP 434
TcSad_XP_970122.2         ---FVKHVFKETLRLYPVAPFLTRILPEDAILGGYGVPKGTLVVMSIYTSGRDGRYFKNP 446
                              :: :.**::****:***: * **::  :  : :  .  :  *:*.:.*:   * :.
                        PERF motif                     Heme-binding
PkSad_variant_B           NQFQPSRWQ-RLENGKYAGVIDPYATLPFAMGARSCIGRNLSKAQLFFTINKIISSFEIE 447
BmSad_NP_001106224.1      DQFLPYRWDRNDQRKKDLVNHVPSATLPFAFGARSCIGKKMAMLQMTELISQIVKNFDLK 454
DmSad_AAL86019.1          ERVLPERWCIGETEQ----VHKSHGSLPFAIGQRSCIGRRVALKQLHSLLGRCTAQFEMS 490
TcSad_XP_970122.2         GLFQPERWD-RKDE-FYSSEMK-KASLPFAMGLRACVGRKVAETQLQMTLLKIVNKFEVE 503
                            . * **     .          .:****:* *:*:*:.::  *:   : :   .*::.

PkSad_variant_B           SLN--EVDVKLNLITLPSQPIHLHLKRRRKTKFKQQQQ 483
BmSad_NP_001106224.1      SMNNSDVDAVTSQVLVPNKDIKVLILPRSISK------ 486
DmSad_AAL86019.1          CLNEMPVDSVLRMVTVPDQTLRLALRPRTE-------- 520
TcSad_XP_970122.2         LGNQREVEIVLKMVAVPSEPLLLKFKKL---------- 531
                            *   *:     : :*.: : : :
```

S1 Fig (continued)

D

```
PkShd                     MIQNFVTTLYNNYSSFRNNKISESYNDQGMYFELTFHIGYSIWITFIVLTGCSIIKRYWK 60
BmShd_NP_001106219.1      -----MSLPGVFLFSHYVESFWSTS-PPLLDWSCVPTLVLAVIAVVVAVT-----ALLTR 49
DmShd_AAQ05972.1          -----MAVILLLALALVLVCYCALHRHKLADIYLRPLLKNTLLEDFYHAE-----LIQPE 50
TcShd_NP_001123894.1      ----------------MFEKIFQS-------LDVTSLLIIAIFFLFLEYR-----PPWWY 32
                                                               :  ::   .

PkShd                     SKTEGKKYPYNIPGPKALPVFGTRWIYSRFG-FYNLNKIHEAYKDLFQRYGNVVKEEALW 119
BmShd_NP_001106219.1      TSDAKHS--CRLPGPQPLPFLGTRW---LFWSRYKMNKLHEAYADMFKRYGPVFMETTPG 104
DmShd_AAQ05972.1          APKRRRRGIWDIPGPKRIPFLGTKWIFLLFFRRYKMTKLHEVYADLNRQYGDIVLEVMPS 110
TcShd_NP_001123894.1      RNNDCKKGVKLIPGPLALPGLGTTWIFF-FG-GFSFNRLHLYYENMYKRYGPVMKEEYWC 90
                               :     :***  :* :** *    *   :.:.::*  * :: ::** :. *

PkShd                     NIPVISIVDKDSIEKVLKQSGKYPFRPPNEVTAYYRKSRPDRYTNLGLVNEQGEVWHHLR 179
BmShd_NP_001106219.1      GVAVVSIAERTALEAVLRSPAKKPYRPPTEIVQMYRRSRPDRYASTGLVNEQGEKWYHLR 164
DmShd_AAQ05972.1          NVPIVHLYNRDDLEKVLKYPSKYPFRPPTEIIVMYRQSRPDRYASVGIVNEQGPMWQRLR 170
TcShd_NP_001123894.1      NIPVINLFEKREIVKVLKAGGKYPLRPPVEAVAHYRRSRPDRYASIGLVNEQGEAWYDLR 150
                          .:.:: : ::  :  **:  .* * *** *    **:******:. *:*****  *  **

PkShd                     LTLTPELTSNKTMKHFLPELTMVTEDFLRLIHTSRNSDNLVID-FEQLCNRLGLESTCTL 238
BmShd_NP_001106219.1      RNLTTDLTSPHTMQNFLPQLNTISDDFLELLNTSRQSDGTVYA-FEQLTNRMGLESVCGL 223
DmShd_AAQ05972.1          SSLTSSITSPRVLQNFLPALNAVCDDFTELLRARRDPDTLVVPNFEELANLMGLEAVCTL 230
TcShd_NP_001123894.1      STLTPALTSPKTITSFLPEAQQIADDWCNLLKLSRDKNGRVSN-LNYIADRLGLELTCAL 209
                           .**. :** :.:  ***    : :*: .*:.  *: :  *   :: : : :*** .* *

PkShd                     VLGKRFGFLQESNVDP-MGKTLADAIRGQFCASRDTFYGLPLWKVFSTPAYKQFIHCEDT 297
BmShd_NP_001106219.1      MLGSRLGFLERWMSG--RAMALAAAVKNHFRAQRDSYYGAPLWKFAPTALYKTFVKSEET 281
DmShd_AAQ05972.1          MLGRRMGFLAIDTKQPQKISQLAAAVKQLFISQRDSYYGLGLWKYFPTKTYRDFARAEDL 290
TcShd_NP_001123894.1      VLGRRMGFLLPGAETE-TGEKLAEAVRQHFLGTRDTYFGFPFWKLFPTPAYKTLIKSEAS 268
                          :** *:***            ** *::  * . **:::*  :**  .*  *: : :.*

PkShd                     IYDIISVLVENAIQEDAELCAV-----DNVHRVFKSILLAPSLDIRDKKAAIIDFIAAGI 352
BmShd_NP_001106219.1      IHAIVTELME---EAKSKTTGMAQDEA--IQEIFLKILENPALDMRDKKAAIIDFITAGI 336
DmShd_AAQ05972.1          IYDVISEIIDHELEELKKSAACEDDEAAGLRSIFLNILELKDLDIRDKKSAIIDFIAAGI 350
TcShd_NP_001123894.1      IYELALELINSANESTKES------------AVFQSVIQA-EIDEREKIAAIIDFISAGI 315
                          *: :   :::   :   :              :* .::    :* *:* :******:***

PkShd                     KTLGNTLVFLLYLVAKHPDVQKKLYQEIQNIISANGTITMDNLRGAVYLRSCIMEAFRLL 412
BmShd_NP_001106219.1      ETLANSLVFLLYLLSGRPDWQRKINSELPPY----AMLCSEDLAGAPSVRAAINEAFRLL 392
DmShd_AAQ05972.1          ETLANTLLFVLSSVTGDPGAMPRILSEFCEYR--DTNILQDALTNATYTKACIQESYRLR 408
TcShd_NP_001123894.1      HTMKNSLLFLLHLIGQDLQIQKKIIEDST----------------KSYSKACVTETFRLL 359
                          .*: *:*:*:*  :        :: .:                      ::.: *::**
                                                                    PERF motif
PkShd                     PTTTCVARILEQKTDINGFNLPPGSVVLCHTWLACLKESNFEKAAEFLPERWLDDVNHKF 472
BmShd_NP_001106219.1      PTAPFLARLLDSPMTTGGHKIPPGTFVLAHTAAACRREENFWRAEEYLPERWIKVQEP-- 450
DmShd_AAQ05972.1          PTAFCLARILEEDMELSGYSLNAGTVVLCQNMIACHKDSNFQGAKQFTPERWIDPATENF 468
TcShd_NP_001123894.1      PTANALGRILEEEMELGGYRLSAGTVVVCHFGIACRDERNFPDASKFKPERWLDDDKVQT 419
                          **:  :.*:*:.    .*. : .*:.*:.:   **  : **  * :: ****:.
                                        Heme-binding
PkShd                     S------CLVIPFGYGRRMCPGKRFVELELQVVLAQIIQNYEIDFNGELELEFEFLLAPA 526
BmShd_NP_001106219.1      ----HAYSLVAPFGRGRRMCPGKRFVELELHLLLAKIMQKWRVEFDGELDIQFDFLLSAK 506
DmShd_AAQ05972.1          TVNVDNASIVVPFGVGRRSCPGKRFVEMEVLLLLAKMVLAFDVSFVKPLETEFEFLLAPK 528
TcShd_NP_001123894.1      AT--NSLFLLTPFGAGRRICPGKRFIEHILPLLLESTVNSFEIQAEEELELQFEFLVTPK 477
                                  :: *** *** ******:*  : ::* . :  : :.    *: :*:**::.

PkShd                     PNTNFIFRNRL- 537
BmShd_NP_001106219.1      SPVTLRLVEW-- 516
DmShd_AAQ05972.1          TPLSLRLSDRVF 540
TcShd_NP_001123894.1      GQMPMVFKDRV- 488
                              : : :
```
