# Supplementary material for: Sex-specific expression profiles of ecdysteroid biosynthesis and ecdysone response genes in extreme sexual dimorphism of the mealybug Planococcus kraunhiae (Kuwana)
Source: PLoS One. 2020 Apr 13;15(4):e0231451. doi: 10.1371/journal.pone.0231451 (PMC7153872; doi:10.1371/journal.pone.0231451)
Supplement: S2 Fig — BgEcR, Blattella germanica EcR (accession number, CAJ01677.1); TcEcR, Tribolium castaneum EcR-A (NP_001107650.1); NvEcR, Nezara viridula EcR-A (ADQ43370.1); PkEcR, Planococcus kraunhiae EcR (this study). Asterisks indicate fully-conserved amino acid residues, while colons and periods represent conservation with strong and weak similarity, respectively. The DNA binding domain (C region) and ligand binding domain (E region) are boxed. The putative junction between EcR-A and EcR-B isoforms is shown by an arrow. (PDF) [file pone.0231451.s002.pdf]

S2 Fig

```
BgEcR_CAJ01677.1        MDLKHEIIYRVGGGGGGGAGTIMKAGLGGLGGGGASGNCLSEELLLSSVK 50
TcEcR_NP_001107650.1    ---------------------MTAICSGNLGSMDIKHEMIYREDDVLLVK 29
NvEcR_ADQ43370.1        --------------------------------------------------
PkEcR                   ------------------------------GG---------RPKGQSVIK 11

BgEcR_CAJ01677.1        TEPRVHSPCDPNTAPATPGVDLGHHLTSSATATSGVTPSGSGGGSAATTP 100
TcEcR_NP_001107650.1    SEPQLHSPCATGSNGLPAGAGSNLLCASNGLVTTLASGSSLNGLTNNVPS 79
NvEcR_ADQ43370.1        --------------------------------------------------
PkEcR                   SEPRSRSPCYNQGREIIR--SPPTASSSSGLFNGIASSKRLRPPDDWLTV 59

BgEcR_CAJ01677.1        NGGS---LFSGIT---SSNKRPRPDDWLSSPSPGSAGPLPLTPSPGPPG 144
TcEcR_NP_001107650.1    NGLSSSGPFSSIGGGISASKRQRTDDWLSSPS----GNVPPLTPSPGPPS 125
NvEcR_ADQ43370.1        -----------------MCEESRRDEWTVGGG------------------ 15
PkEcR                   TPRSPVTVVSSVA-----VSASTSTNLSSSPG-------------PALHS 91
                                           .     :     .  .

BgEcR_CAJ01677.1        HPYTVISNGYSSP-MSSGSYDPYSPNGKLGREDLSPQSSLNGHSFDGCD- 192
TcEcR_NP_001107650.1    HPYTVISNGYSSP-MSSGSYDPYSPNGKIGREDLSP-SSLNGYSADSCD- 172
NvEcR_ADQ43370.1        ------QNGCPSPTMSSNSYEPYSPASKLGREDLSPANSLNGFSADSCDG 59
PkEcR                   SIYPSTSNGYPSPTMSTNSYDLYNSGSKLSGEDLSP-NSQNGYMMEVCDA 140
                              .** .** **:.**: *.. .*:. ***** .* **.  : **

BgEcR_CAJ01677.1        AKKKKGPAP--RQQEELCLVCGDRASGYHYNALTCEGCKGFFRRSITKNA 240
TcEcR_NP_001107650.1    SKKKKGPTP--RQQEELCLVCGDRASGYHYNALTCEGCKGFFRRSITKNA 220
NvEcR_ADQ43370.1        SKKKKGSAA--RQQEELCLVCGDRASGYHYNALTCEGCKGFFRRSITKNA 107
PkEcR                   SKKKKGPTATTRQQEELCLVCGDRASGYHYNALTCEGCKGFFRRSITKNA 190
                        :*****.:.  **************************************
C region
BgEcR_CAJ01677.1        VYQCKYGNNCEIDMYMRRKCQECRLKKCLSVGMRPECVVPEYQCAVKRKE 290
TcEcR_NP_001107650.1    VYQCKYGNNCEIDMYMRRKCQECRLKKCLSVGMRPECVVPEVQCAVKRKE 270
NvEcR_ADQ43370.1        VYQCKYGNNCEIDMYMRRKCQECRLKKCLSVGMRPECVVPEYQCAVKRKE 157
PkEcR                   VYQCKYGNHCDIDMYMRRKCQECRLKKCLSVGMRPECVVPEYQCAVKRKE 240
                        ********:*:******************************** ********

BgEcR_CAJ01677.1        KKAQKDKDKPNSTTNGSPEVVMMKETGTKVEPEK------LPTVNGVKPV 334
TcEcR_NP_001107650.1    KKAQKEKDKPNSTTNGSPDVIKVEPELSDSEK---------TLCNGSKGI 311
NvEcR_ADQ43370.1        KKAQKDKDKPVSTTNGSPESIKVEEPHRVVYKTEGETLGRPVPINGVKPV 207
PkEcR                   KKAQKDKDKPNSTTNGSPETIKIEPELQRVDSDP-------MMVNGVKPV 283
                        *****:**** *******: : ::                 ** * :
                                                       E region
BgEcR_CAJ01677.1        SPEQEELITRLVYFQNEYEQPSEEDLKRITN-QPTEGEDQSDLKFRHITE 383
TcEcR_NP_001107650.1    SPEQEELIHRLVYFQNEYEHPSEEDVKRIIN-QPMDGEDQCDVRFRHITE 360
NvEcR_ADQ43370.1        SPEQEELIHRLVYFQNEYEHPSEEDLRRIVSNTPNEDEEQSDQRFRHLTE 257
PkEcR                   STEQEELIHSLVYFQEQYEQPSPDDLQRVFSSDETDPHAEGDMKYKHITE 333
                        *.******  *****::**:** :*:*:*: .      : .  : * ::*:**

BgEcR_CAJ01677.1        ITILTVQLIVEFAKRLPGFDKLLREDQIALLKACSSEVMMFRMARRYDVN 433
TcEcR_NP_001107650.1    ITILTVQLIVEFAKRLPGFDKLLREDQIALLKACSSEVMMFRMARRYDVQ 410
NvEcR_ADQ43370.1        ITILTVQLIVEFAKRLPGFDKLLREDQIALLKACSSEVMMLRMARKYDAT 307
PkEcR                   MTILTVQLIVEFAKSLPGFDKLLREDQITLLKACSSEVMMLRMARKYDSN 383
                        :************* *************:***********:****:**

BgEcR_CAJ01677.1        SDSILFANNQPYTRDSYSLAGLGETIEDMLRFCRQMYAMKVDNAEYALLT 483
TcEcR_NP_001107650.1    TDSILFVNNQPYSRDSYNLAGMGETIEDLLHFCRTMYSMKVDNAEYALLT 460
NvEcR_ADQ43370.1        TDSILFANNQPYNRESYSLAGMGDVIEDLLRFCRHMFNMKVDNAEYALLT 357
PkEcR                   TDSIVFANNQPYTKDSYNDAGMGETIEDLLRFCRQMWAMKVDNAEYALLT 433
                        :***:*.*****.::**. **:*:.***:*:*** *: ************

BgEcR_CAJ01677.1        AIVIFSDRPSLIEGWKVEKIQEIYLEALKAYVDNRRRPRSGTIFAKLLSV 533
TcEcR_NP_001107650.1    AIVIFSERPALIEGWKVEKIQEIYLEALRAYVDNRRKPKPGTIFAKLLSV 510
NvEcR_ADQ43370.1        AIVIFSERPALIEGWKVEKIQEIYLEALKSYVDNRSRPRSPTIFAKLLSV 407
PkEcR                   AIVIFSGRPSLVEDWKVEKIQEVYIETLKSYVDNRVRPNSTTIFARLLSV 483
                        ****** **:*:*.********:*:*:*::***** :*.. ****:****

BgEcR_CAJ01677.1        LTELRTLGNQNSEMCFSLKLKNKELPPFLAEIWDLTP-- 570
TcEcR_NP_001107650.1    LTELRTLGNQNSEMCFSLKLKNKKLPPFLAEIWDVDLKT 549
NvEcR_ADQ43370.1        LTELRTLGNQNSEMCFSLKLQNKKLPPFLAEIWDVNT-- 444
PkEcR                   LTELRTLGNRNFEMCLTLKLKSKVLPPFLSEIWDVAS-- 520
                        *********:* ***::***:.* *****:****:
```
