# Supplementary material for: Sex-specific expression profiles of ecdysteroid biosynthesis and ecdysone response genes in extreme sexual dimorphism of the mealybug Planococcus kraunhiae (Kuwana)
Source: PLoS One. 2020 Apr 13;15(4):e0231451. doi: 10.1371/journal.pone.0231451 (PMC7153872; doi:10.1371/journal.pone.0231451)
Supplement: S3 Fig — PkE75A, Planococcus kraunhiae E75 isoform A (this study); BgE75A, Blattella germanica E75A (accession number, CAJ87513.1); BmE75A, Bombyx mori E75A (NP_00106079.1). Asterisks indicate fully-conserved amino acid residues, while colons and periods represent conservation with strong and weak similarity, respectively. The DNA-binding domain and ligand-binding domain are boxed. (PDF) [file pone.0231451.s003.pdf]

S3 Fig

```
PkE75A                    MFLYEGTDIRKATSYEYEFTKSHTKSDFNGNFDIYDGDYMDDYEKKQNEP 50
BgE75A_CAJ87513.1         ---------MIVTESAPYEMMQEHLQLPESTTPGIMMDNTVTSVHR---EP 39
BmE75A_NP_001106079.1     ----------MSPDSSYGRYDVPTSVDHS---------LMSSMHKER-EP 30
                                     : .  *   .   .   .         : . .:   **

                                DBD
PkE75A                    KLNLEPDGTTVLCRVCGDKASGFHYGVHSCEGCKGFFRRSIQQKIQYRPC 100
BgE75A_CAJ87513.1         ELKIEPDGTTVLCRVCGDKASGFHYGVHSCEGCKGFFRRSIQQKIQYRPC 89
BmE75A_NP_001106079.1     ELHIEPDGTTVLCRVCGDKASGFHYGVHSCEGCKGFFRRSIQQKIQYRPC 80
                          :*::**********************************************

PkE75A                    TKNQQCSILRINRNRCQYCRLKKCIAVGMSRDAVRFGRVPKREKARILAA 150
BgE75A_CAJ87513.1         TKNQQCSILRINRNRCQYCRLKKCIAVGMSRDAVRFGRVPKREKARILAA 139
BmE75A_NP_001106079.1     TKNQQCSILRINRNRCQYCRLKKCIAVGMSRDAVRFGRVPKREKARILAA 130
                          **************************************************

PkE75A                    MQQSTNSRSQEKALTAELEDDQRLLATVVRAHLETCDFTRDKVEPMLIRA 200
BgE75A_CAJ87513.1         MQQSSNSRSQEKAVAAELEDEQRLLSTVVRAHLDTCDFTREKVEPMLARA 189
BmE75A_NP_001106079.1     MQQSSSSRAHEQAAAAELDDAPRLLARVVRAHLDTCEFTRDRVASMRARA 180
                          ****:.**::*:*  :***:*   ***:  ******:**:***::*  .*   **

                                                               LBD
PkE75A                    RTQPSFTACPPTLACPLNPNPQPLTGQQELLQDFSKRFSPAIRGVVEFAK 250
BgE75A_CAJ87513.1         RDQPSYTACPPTLACPLNPNPQPLTGQQELLQDFSKRFSPAIRGVVEFAK 239
BmE75A_NP_001106079.1     RDCPTYS--QPTLACPLNPAPELQSEK-----EFSQRFAHVIRGVIDFAG 223
                          *  *:::    ********* *:  : :      :**:**: .****::**

PkE75A                    RITGFALLPQDDQVTLLKAGVFEVLLVRLACMFDAQNNTMICLNGQVLKR 300
BgE75A_CAJ87513.1         RIPGFALLPQDDQVTLLKAGVFEVLLVRLACMFDAQTNSMICLNGQVLKR 289
BmE75A_NP_001106079.1     LIPGFQLLTQDDKFTLLKSGLFDALFVRLICMFDAPLNSIICLNGQLMKR 273
                           *.** **.***:.****:*:*:*:.*:**** *****   *::******::**

PkE75A                    DSIHNSSNARFLMDSMFDFAERLNSLHLSDAEIGLFSSIVVIAADRPGLR 350
BgE75A_CAJ87513.1         EAIHNSSNARFLMDSMFDFAERLNSLRLSDAEVGLFCSVVVIAPDRPGLR 339
BmE75A_NP_001106079.1     DSIQSGANARFLVDSTFKFAERMNSMNLTDAEIGLFCAIVLITPDRPGLR 323
                          ::*:..:*****:** *.****:**:.*:***:***.::*:*:.******

PkE75A                    NVELIEKMHNKLKALLQSVLAQNHPTQPSLCQELIKKIPDLRTLNTLHSE 400
BgE75A_CAJ87513.1         NTELIERMQGKLKAALQMVVSQNHPGHANICHELMKKIPDLRTLNTLHSE 389
BmE75A_NP_001106079.1     NIELVERMHSRLKACLQAVIAQNRPERPGFLRELMDTLPDLRTLSTLHTE 373
                          * **:*:*:.:*** ** *::**:*  :..: :**:..:******.***:*

PkE75A                    KLLAFKMTEQQQLQQQHNLWNGSNGAVKYKQEPIDYHRGLDDCGGTKSPV 450
BgE75A_CAJ87513.1         KLLAFKMTEQQQLQQQ-------------QQQHLWGTSPEEESNSKSPA 426
BmE75A_NP_001106079.1     KLVVFRTEHKELLRQQMWN---------------------EEEG------ 396
                          **:.*:  .:: *:**                           :: .

PkE75A                    GSSSWSSSSEGGAMEEVKSPMGSVSSTESACSGEVAALNENAAAVASIVS 500
BgE75A_CAJ87513.1         GSSSWSSSSDVTMDEAVKSPLGSVSSTESVCSGEVASLTEYQPNHHPVS- 475
BmE75A_NP_001106079.1     --VSWADS---VVEESARSPSGSVSSSE---SGEVPS------------- 425
                             **:.*      * .:** *****:*   ****.:

PkE75A                    GHQAANAPLLAATLAAGVCPMRRRQNSIGSMSGDDDATNAASLHAHPHLH 550
BgE75A_CAJ87513.1         -HQASSAPLLAATLAGGICPHRHRANS-GSTSGDDDMS-GLPHHSHHGLT 522
BmE75A_NP_001106079.1     ---DCGTPLLAATLAG-----RRRLDS---RGSVDEEALGVAHLAHNGLT 464
                              ..:********.     *:* :*    .. *: : . .  :*  *

PkE75A                    MKLQQQQQQHHQLQLHHHHQAAAAQQHLVSHKSTFPRKLDSPCDSGIES 600
BgE75A_CAJ87513.1         ITAVNPPSRQQPLMPQHHHR----------------FQRKLDSPSDSGIES 557
BmE75A_NP_001106079.1     VTPVRPPPR----------------------------YRKLDSPTDSGIES 487
                          :.   .   :                                ****** ******

PkE75A                    GTEKIEKLTN------SAPTSVCSSPRSSLEDKDEHNHHQQHHHHHQHH 644
BgE75A_CAJ87513.1         GTEKLDKLTSGGGSTGSAPTSVCSSPRSSLEDKDEEKHHN---------- 597
BmE75A_NP_001106079.1     GNEKHERIIG--------PGSGCSSPRSSLEEHTEDRRPT---------- 519
                          *.** ::: .           * * *********:: *..:

PkE75A                    HHNSNSSSIHHNGNQSVNNQQNGGSSASAGQSIDDMPVLKRVLQAPPLYD 694
BgE75A_CAJ87513.1         ------------------------GSSGSS--HIDDMPVLKRVLQAPPLYD 622
BmE75A_NP_001106079.1     ---------------------------------APADDMPVLKRVLQAPPLYG 539
                                                                 ***************.
```

S3 Fig (continued)

```
PkE75A                     -TNSLMDEAYKPHHKKFRALRNKDTAEAEPIVVVT-----------HNGS 731
BgE75A_CAJ87513.1          -TNSLMDEAYKPHHKKFRACRNKDSAEAEPMIVHVSPPPPSHPVPPQHHSS 671
BmE75A_NP_001106079.1      GTSTLMDETYKPHHKKFRAMR-RDTGEAEARPVQP-----------TPSP 576
                            *.:****:********** * :*:.***.  *               ..

PkE75A                     LSGGNSSSSGNQPQSQLQHHLTSSLSSTHSTLAKSLMEGPRMTPEQMKRT 781
BgE75A_CAJ87513.1          PQLHLHLTSNNHQSQSSTSSTSSSLSSTHSTLAKSLMESPRMTAEQLKRT 721
BmE75A_NP_001106079.1      QPLHPHPASPAHPAHS-PRPPRISLSSTHSVLAKSLMEGPRMTPEQLKRT 625
                                  :*  :   .       *******.*******.****.**:***

PkE75A                     DIIHNFIMRG------ADALESTSQQSSSGSSSGSSTSS----------- 814
BgE75A_CAJ87513.1          DIIHNYIMRADSPNPVTIEFPPSAPSPSANSASTTSTSSYKMNSGNLLVC 771
BmE75A_NP_001106079.1      DMIQQYMRRN---------------------EAGSSVEG----------- 643
                           *:*:::: *                       : :*...

PkE75A                     -RCSPNQQIYYVP--QPTKWEQNGVAGELSTGAGTIGVGTSVITSSGRPQ 861
BgE75A_CAJ87513.1          ANSTPSTGYHYIPQIQPQQQQQQTVVGRWQGSPGFSNGASVITTTTGRNP 821
BmE75A_NP_001106079.1      ---------------------------------CPLRTGGLLTCYRGASP 660
                                                                 .  :    *

PkE75A                     VSSSPYLNTVVRSQSLSP---HGEP----ITKIYLR-GTNLSPPTCLSSS 903
BgE75A_CAJ87513.1          TPQQTYV-LLQNSTNISPPVHHAEMQQSEFSRIYFHPGNAVSPHHAAASS 870
BmE75A_NP_001106079.1      AP----------------------------------------PPVLALQV 670
                           ..                                        *     .

PkE75A                     S---SPVHFLHSQSP-GLSQSPPMHVQS---------------------- 927
BgE75A_CAJ87513.1          STSPSPLPIPHRKTPPAVASCPPSPSSSGVTPIVISSPKMMELQVDIADS 920
BmE75A_NP_001106079.1      DVTDAPLNLSKKSPSPPRSYMPQMLEA----------------------- 697
                           .   :*: : : ...   :  *

PkE75A                     ----------------------------------
BgE75A_CAJ87513.1          QQPLNLSKKSPSPSPHPMASPATTVTHKVVSLEA 954
BmE75A_NP_001106079.1      ----------------------------------
```
