# Supplementary material for: Sex-specific expression profiles of ecdysteroid biosynthesis and ecdysone response genes in extreme sexual dimorphism of the mealybug Planococcus kraunhiae (Kuwana)
Source: PLoS One. 2020 Apr 13;15(4):e0231451. doi: 10.1371/journal.pone.0231451 (PMC7153872; doi:10.1371/journal.pone.0231451)
Supplement: S1 Table — (PDF) [file pone.0231451.s004.pdf]

S1 Table

	Spook (CYP307A1)	Phantom (CYP306A1)	Disembodied (CYP302A1)	Shadow (CYP315A1)	Shade (CYP314A1)
Nilaparvata lugens	XP_022199324.1	AIW79976.1	-	XP_022207767.1	AIW79959.1
Laodelphax striatella	AFU86444.1	AGI92300.1	-	AGU16451.1	AGI92301.1
Sogatella furcifera	AGI92294.1	AQM57053.1	-	AGI92297.1	AGI92296.1
Bemisia tabaci	XP_018915691.1	XP_018918264.1	XP_018916455.1	XP_018917274.1	XP_018900763.1
Tribolium castaneum	EFA11558.2	XM963384	XP_974252.1	XP_970122.2	NP_001123894.1
