# Supplementary material for: Sex-specific expression profiles of ecdysteroid biosynthesis and ecdysone response genes in extreme sexual dimorphism of the mealybug Planococcus kraunhiae (Kuwana)
Source: PLoS One. 2020 Apr 13;15(4):e0231451. doi: 10.1371/journal.pone.0231451 (PMC7153872; doi:10.1371/journal.pone.0231451)
Supplement: S2 Table — (PDF) [file pone.0231451.s005.pdf]

S2 Table

Primer name	Sequence (5'–3')
**RT-PCR**	
PkSpook-F1	AGACCGCAACGTGATCTACC
PkSpook-R1	TTAGCTGTAGGACAGCCTTACGA
PkDib-F1	GAATTTGGTCCTCTGGTACGC
PkDib-R1	TGCATGTACCCGAGTAAAGTAGG
PkShadow-F1	CATTCTCTGTTCCTCTGCTTCC
PkShadow-R1	CGATGCACATTATTGTTGCTG
PkShade-F1	TTCATCGGACCGGAAAGATA
PkShade-R1	TCGGCTTTGATAGGTGGATG
PkEcR-F1	ATGGAGGCAGGCCGAAAGGTCAGTC
PkEcR-R1	TTAAGAAGCAACGTCCCAGATTTC
PkE75-1-F1	AACACTCATATCGCTCAAGAGG
PkE75-2-F1	ATGAGCCAAAGCTTAACTTG
PkE75-F2	CTCCTACTTTGGCTTGTCCACTGA
PkE75-F3	CATCATTATCCGGTCATCCAGC
PkE75-F4	CCGGCAGCAACTTCTCAGAT
PkE75-R1	AGGGCGTCGGCTCCTCGCAT
PkE75-R2	CTTTTCGATTTTCTCGGTGCCGGA
PkE75-R3	GTTGGGTTCGAGCTCTAATC
PkE75-R4	TCTGACCACAGTGGCTAATA
**5'RACE PCR**	
PkE75-RR1	GAATACGCGCTTTTTCTCGTTTCGGTACGC
PkE75-1-RR2	TGGACAAGCCAAAGTAGGAGGACAAGCGGT
PkE75-1-RR3	TCCTGATGGGCGACAAAGTTGCGGGA
**3'RACE PCR**	
PkE75-RF1	TACCCATAACGGTAGCTTGAGCGGCGGC
PkE75-RF2	TCGTACGTAGTCAATCGTTA
PkE75-RF3	ACGTACCTCAGCCGACCAAATGGGAAC
PkE75-RF4	CCAAGATATATCTACGCGGT
**qRT-PCR**	
PkSpook-QF1	CGAATCGAAGAGGAAATCCA
PkSpook-QR1	AAAACGGTCGCCTCAGTGTA
PkDib-QF1	AAATCCCAGTGCTGCTCAAC
PkDib-QR1	AGCAGATCAGCAGCCATACC
PkShadow-QF1	ATTCGGCCCGATTTTCTTAG
PkShadow-QR1	GTTTCGGATAGCGTCCTTCA
PkShade-QF1	ACTTTGGTGCTTGGCAAAAG
PkShade-QR1	TGCACAAAATTGTCCTCGAA
Pkrp49-QF2	CCATCAAAGTGACCGCTATGTC
Pkrp49-QR2	CGCGTTACTTCCATATCCAACA
PkEcR-QF1	TGGAGGTTTGCGATGCCAGT
PkEcR-QR1	ACCCTGAGGCCCTATCACCA
PkE75A-QF1	TTCGGTGCTAATGTGTCTGGGT
PkE75A-QR1	TGGCACCAAAAATTCAAGCACG
PkE75B-QF1	GGCAATTGCAAGGACGCTGATT
PkE75B-QR1	TTTCCATATCGGGCATAGGGGG
PkE75C-QF1	TCTTCAACCTGTTCCGATGCGA
PkE75C-QR1	TTGGATTGTATGGGCTGCTGGA
PkE75D-QF1	TGTGAACGAATTTCGCGCCTTA
PkE75D-QR1	TACAGCCACACACTTGTCACGT
PkE75E-QF1	GATGCGTTCGTGTCGTTGAAGT
PkE75E-QR1	ACCAATTGTTGACGACTAGATCACA
PkE75com-QF1	CCGCGCAACAGCATCTAGTTAG
PkE75com-QR1	TTTCTCGGTGCCGGATTCGATA
